# α-catenin phosphorylation is elevated during mitosis to resist apical rounding and epithelial barrier leak

**DOI:** 10.1242/bio.061726

**Published:** 2025-01-08

**Authors:** Phuong M. Le, Jeanne M. Quinn, Annette S. Flozak, Adam W. T. Steffeck, Che-Fan Huang, Cara J. Gottardi

**Affiliations:** ^1^ Department of Pulmonary Medicine, Northwestern University Feinberg School of Medicine, Chicago, IL 60611, USA; ^2^ Driskill Graduate Program in the Life Sciences, Northwestern University Feinberg School of Medicine, Chicago, IL 60611, USA; ^3^ Proteomics Center of Excellence, Northwestern University, Evanston, IL 60208, USA; ^4^ Cell & Developmental Biology, Northwestern University Feinberg School of Medicine, Chicago, IL 60611, USA

**Keywords:** Adherens junctions, Epithelial barrier, Tight junctions, Cadherins, Catenins, Mitosis

## Abstract

Epithelial cell cohesion and barrier function critically depend on α-catenin, an actin-binding protein and essential constituent of cadherin-catenin-based adherens junctions. α-catenin undergoes actomyosin force-dependent unfolding of both actin-binding and middle domains to strongly engage actin filaments and its various effectors; this mechanosensitivity is critical for adherens junction function. We previously showed that α-catenin is highly phosphorylated in an unstructured region that links the mechanosensitive middle and actin-binding domains (known as the P-linker region), but the cellular processes that promote α-catenin phosphorylation have remained elusive. Here, we leverage a previously published phospho-proteomic data set to show that the α-catenin P-linker region is maximally phosphorylated during mitosis. By reconstituting α-catenin CRISPR knockout MDCK cells with wild-type, phospho-mutant and phospho-mimic forms of α-catenin, we show that full phosphorylation restrains mitotic cell rounding in the apical direction, strengthening the interactions between dividing and non-dividing neighbors to limit epithelial barrier leak. As the major scaffold components of adherens junctions, tight junctions and desmosomes are also differentially phosphorylated during mitosis, we reason that epithelial cell division may be a tractable system to understand how junction complexes are coordinately regulated to sustain barrier function under tension-generating morphogenetic processes.

## Teaser

**Summary:** Adherens junction regulation during cell division relies on force-dependent unfolding of the mechanosensitive scaffold protein α-catenin. Here, we show that α-catenin modification by phosphorylation also increases during mitosis to reinforce epithelial barrier function.

## INTRODUCTION

Simple epithelia comprise a single layer of cells organized into sheets, where they form versatile barriers that compartmentalize tissue organization and functions across organ systems. A key feature that allows individual epithelial cells to form such barriers are intercellular adhesive junctions, which coordinate the coupling of cytoskeletal networks across cells (via adherens junctions and desmosomes), and passage of small-molecule constituents between apical and basolateral compartments (via tight junctions) (Angulo-Urarte et al., 2020; Broussard et al., 2020; Citi, 2019; Yap et al., 2018). As organismal development initiates from the expansion and rearrangement of cells within epithelial sheets, and environmental insults can activate epithelial repair programs, a key question in the field is how cell-cell junction complexes are regulated to allow for dynamic cell-cell behaviors while maintaining overall barrier integrity (Higashi et al., 2024). Indeed, a major challenge in understanding cell-cell adhesion regulation is identifying a well-defined morphogenetic process for which complementary proteomic data are also available.

Epithelial cell division is emerging as an ideal system to understand cell-cell junction regulation, as cells dividing in an epithelium undergo defined membrane shape changes, such as apically directed rounding and retraction from the basement membrane to accommodate the mitotic spindle (McKinley et al., 2018), to partitioning the cytoplasm via cytokinesis (Derksen and van de Ven, 2020; van de Ven et al., 2016; Wolf et al., 2006) and resolving the midbody through an apical junction abscission mechanism (Bai et al., 2020; Daniel et al., 2018; Herszterg et al., 2014; Higashi et al., 2016; Morais-de-Sa and Sunkel, 2013a,b). In vertebrate systems, this entire sequence occurs with continuous connection of adherens and tight junction constituents to the actomyosin contractile ring during cytokinesis and full maintenance of the transepithelial barrier (Higashi et al., 2016), suggesting that epithelial junctions can withstand mitotic forces.

Recent studies suggest that adherens junctions, particularly the cadherin-catenin adhesion complex and its essential actin-binding component α-catenin (α-cat), may be a central mechanosensitive mediator of epithelial cell division. In cleaving *Xenopus* embryos, E-cadherin and β-catenin proteins showed reduced mobility at the cytokinetic furrow relative to that at the non-dividing membrane interface, along with enhanced recruitment of vinculin, a homolog and mechanosensitive binding partner of α-cat (Higashi et al., 2016). Related work in dividing Madin–Darby canine kidney (MDCK) epithelial monolayers revealed that, as a mitotic cell rounds up and away from its neighbors, it generates increased tension on the junctions of an adjacent cell, favoring vinculin recruitment (Monster et al., 2021). This asymmetric recruitment of vinculin to adherens junctions in neighboring rather than dividing cells contributes to epithelial barrier integrity, as MDCK cells reconstituted with an α-cat mutant that cannot recruit vinculin showed clear gaps and barrier leak when present in neighboring rather than mitotic cells. Taken together, these data suggest that the cadherin-catenin complex is mechanically altered during mitosis to promote effector (e.g. vinculin) recruitment to preserve epithelial barrier integrity. Whether adherens junction regulation during cell division largely relies on force-dependent unfolding of α-cat, independently of other modes of regulation, is not known. In this study, we show that α-cat phosphorylation is upregulated during mitosis and contributes to epithelial barrier function in MDCK cells. Along with previously published phospho-proteomic datasets showing that major scaffold components of adherens junctions, tight junctions and desmosomes are differentially phosphorylated during mitosis (Dephoure et al., 2008), we reason that epithelial cell division may be a tractable system to understand how adhesive junction complexes are regulated.

## RESULTS

### α-cat phosphorylation is increased during mitosis

**Fig. 1.**
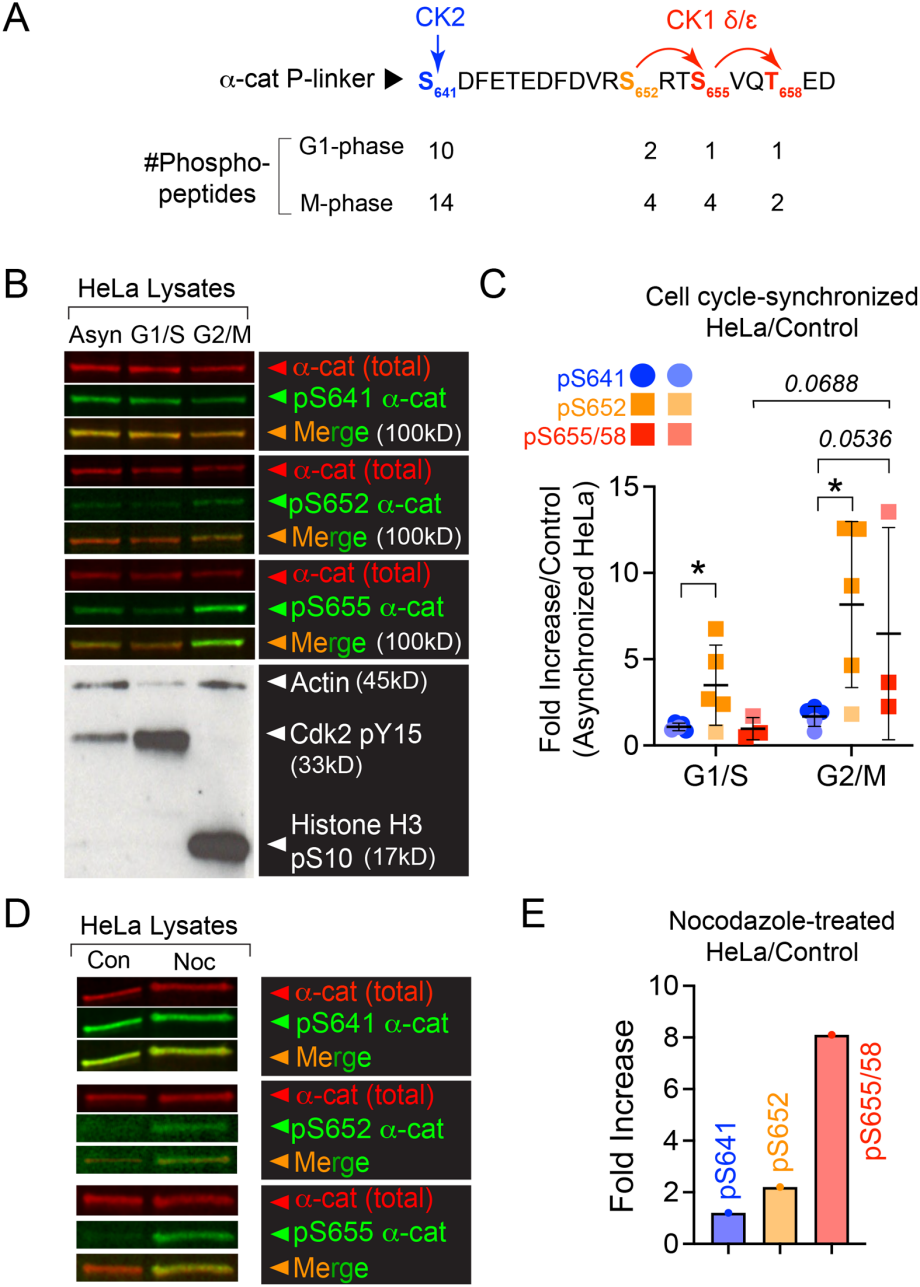
**α-cat phosphorylation is increased during mitosis in cell cycle-synchronized and nocodazole-treated HeLa cell lysates.** (A) Phospho-peptide detection during G1 and mitotic (M) phases of the cell cycle by stable isotope labeling phospho-peptide enrichment mass spectrometry (Dephoure et al., 2008). The schematic shows the previously defined *in vitro* dual-kinase mechanism, wherein casein kinase 2 (CK2)-mediated phosphorylation at S641 primes α-cat for subsequent and sequential phosphorylations by casein kinase 1 (CK1) at S652, S655 and T658 (Escobar et al., 2015). (B) Immunoblot validation of α-cat phosphorylations at S641 (Signalway), S652 and S655/T658 (Cell Signaling Technology) in asynchronized and synchronized G1/S and G2/M HeLa cell lysates. Actin, pY15 Cdk2 and pS10-histone 3 were used as loading controls to validate cell cycle phases. (C) Quantification of α-cat phospho-site detection from multiple immunoblots (symbol) and two independent HeLa cell preparations [Abcam, lots K (dark symbol) and M (light symbol)]. Data are shown as fold increase over the asynchronized HeLa control condition and presented as mean±s.d. with significance between pS641 and pS652 (G1/S) (*P=0.0457) and pS641 and pS652 (G2/M) (*P=0.0173) by unpaired two-tailed Student's *t*-test. The *P*-value for the pS641 versus pS655/T658 comparison (G2/M) is shown in the graph as *P*=0.0536. The *P*-value for the pS655/T658 site in G1/S versus G2/M comparison is shown in the graph as *P*=0.0688. (D) Immunoblot analysis of α-cat phosphorylation using control (Con) and nocodazole-treated (Noc) HeLa cell lysates. (E) Quantification of data shown in D reflects the fold increase in expression in the nocodazole-treated over the control HeLa condition.

Quantitative phospho-proteome profiling of various cell and tissue systems confirmed evidence by our group that α-cat is reproducibly phosphorylated at multiple sites in an unstructured region that links the mechanosensitive middle and actin-binding domains (Ballif et al., 2004; Beausoleil et al., 2004; Dephoure et al., 2008; Escobar et al., 2015; Huttlin et al., 2010; Olsen et al., 2006; Zhai et al., 2008). Although *in vitro* kinase assays using purified recombinant α-cat as substrate established a casein kinase 2 (CK2)-casein kinase 1 (CK1) dual-kinase mechanism (Escobar et al., 2015) (Fig. 1A), upstream signals and processes that regulate α-cat phosphorylation remained elusive. Curiously, stable isotope labeling of HeLa cells arrested in the G1 or mitotic phases of the cell cycle suggest that α-cat phosphorylation is quantitatively increased during mitosis (Dephoure et al., 2008) (Fig. 1A), but reproducibility of this regulation and its role in epithelial cell division are lacking. We used commercially available antibodies that recognize distinct α-cat phospho-sites to immunoblot lysates prepared from HeLa cells synchronized in the G1/S or G2/M phases of the cell cycle (Fig. 1B). We found that α-cat phosphorylation at S641 is not obviously enhanced by mitosis, whereas α-cat phosphorylated at S652 or S655/T658 clearly increases during mitosis (Fig. 1B,C). The antibody against phosphorylated (p)S655/T658 generally performed better (i.e. signal to noise ratio), possibly because it recognizes two phosphorylated residues (S655/T658) to the single residue of pS652. As the increase in α-cat phosphorylation at pS652 and pS655/T658, particularly at the G2/M phase of mitosis, showed variability across two independent HeLa cell cycle-synchronized lysates (∼2.5 to >10-fold), we also interrogated α-cat phosphorylation after nocodazole-treatment, which is known to mimic an actomyosin hypercontractility state through microtubule depolymerization and release of guanine nucleotide exchange factor H1 (GEF-H1), leading to Rho activation (Chang et al., 2008). Interestingly, nocodazole-treated HeLa lysates showed the same trend of increased phosphorylation at pS652 and pS655/T658 (Fig. 1D,E). Collectively, these data suggest that mitotic rounding (induced by cell cycle synchronization or nocodazole treatment) does not impact α-cat phospho-priming at the most abundant site (S641), but rather increases phosphorylation at previously defined CK1 sites (pS652, pS655/T658), which are sequentially related (Escobar et al., 2015). As this phospho (P)-domain resides in a region that links the middle and actin-binding domains, we refer to this as the P-linker region (Escobar et al., 2015).

### Phospho-mimic α-cat restrains mitotic apical rounding

**Fig. 2.**
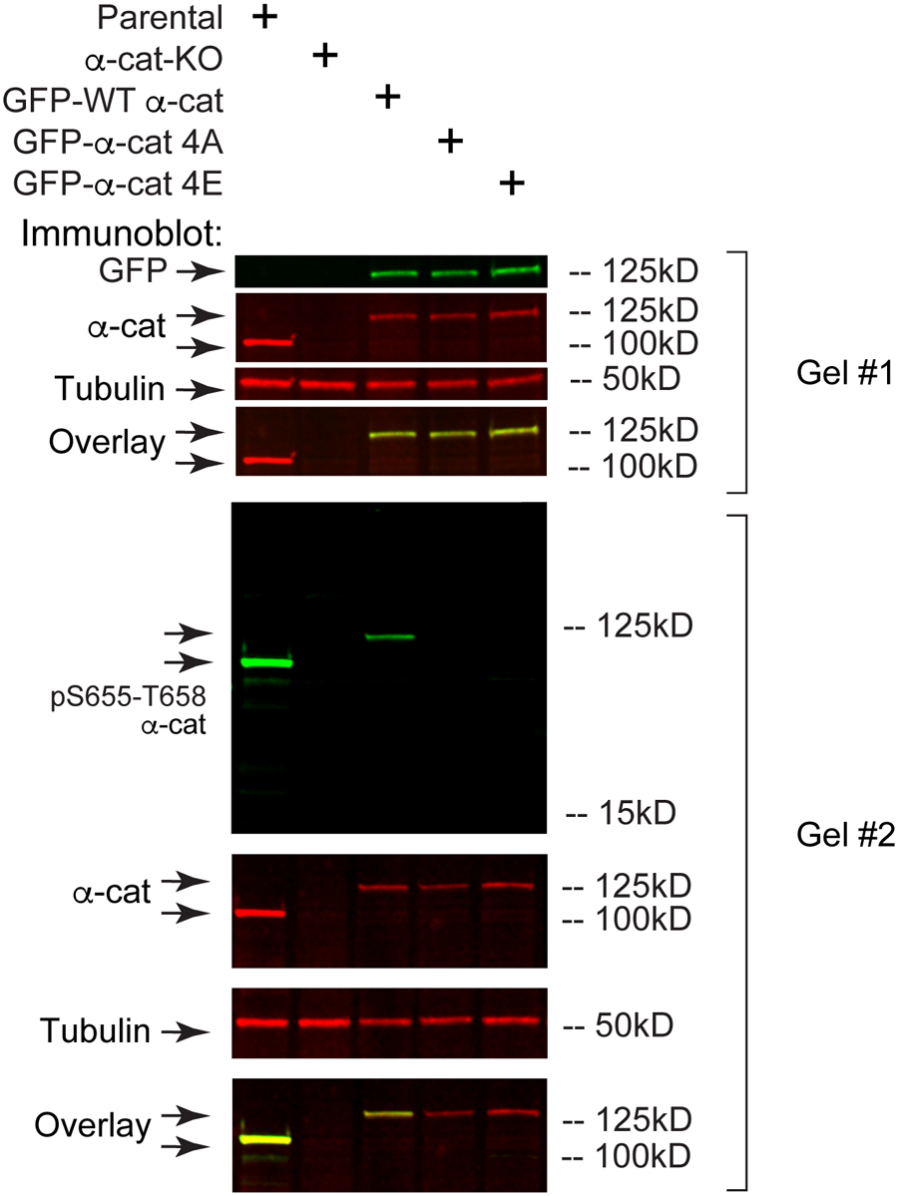
**GFP-α-cat mutants express α-cat similarly in α-cat knockout MDCK cells.** Immunoblot validation of α-cat and GFP-α-cat in parental, α-cat-knockout (KO), α-cat-KO^GFP-α-cat-WT^, α-cat-KO^GFP-α-cat-4A^ and α-cat-KO^GFP-α-cat-4E^ MDCK cell lines using antibodies specified in the Materials and Methods. Tubulin was used as a loading control to validate loading protein amount (gel #1). Antibodies to α-cat phosphorylated at S655/T658 do not recognize α-cat-4A or -4E mutant constructs, as expected (gel #2). Images represent >20 independent immunoblotting experiments.

**Fig. 3.**
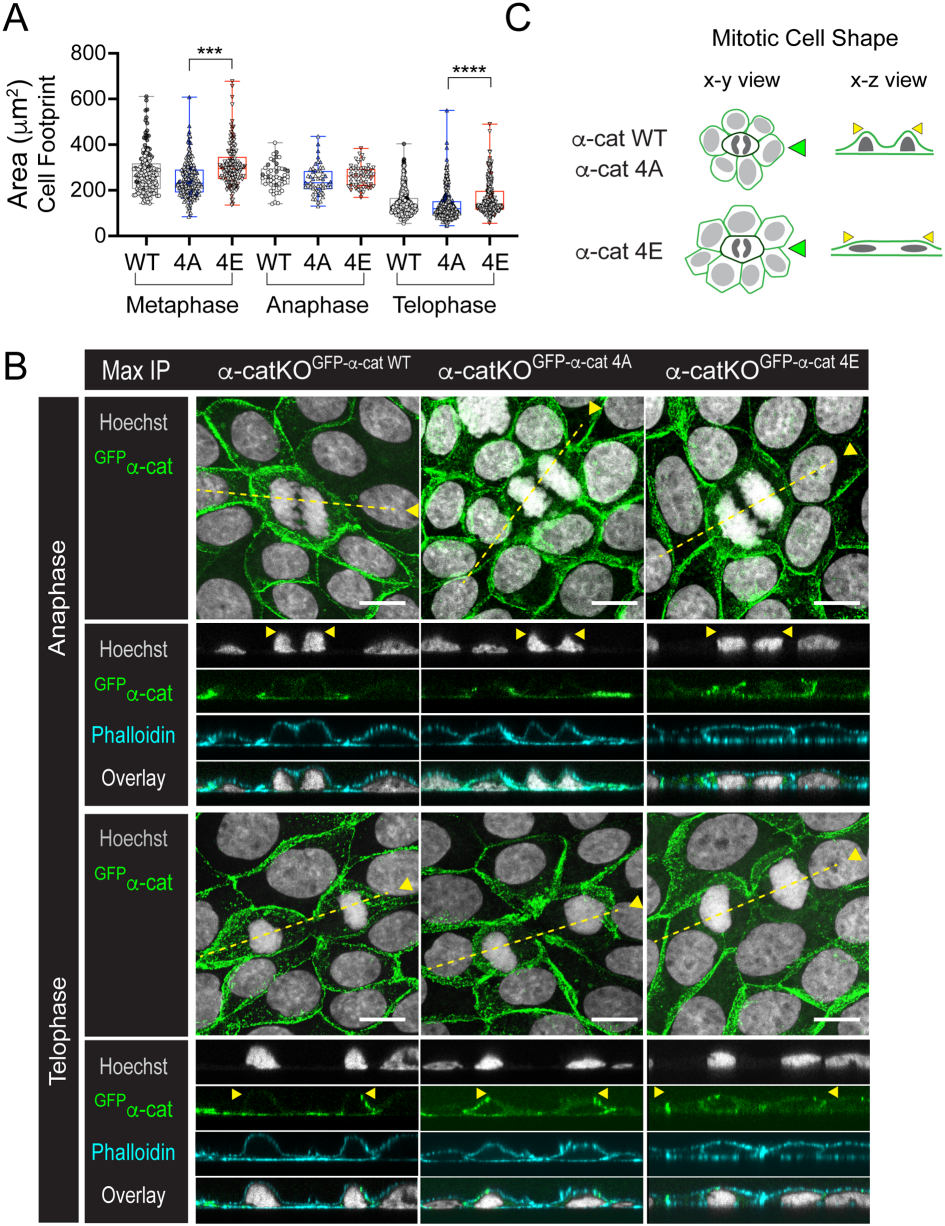
**Phospho-mimic α-cat restrains mitotic rounding compared with WT and phospho-mutant α-cat.** (A) Quantification of cell area (μm^2^) of α-cat cell lines during mitotic phases. Data are presented as mean±s.d. with significance determined by one-way ANOVA with Kruskall–Wallis test (****P<0.0001; ***P=0.0003). Images were captured using a 20× objective Nikon Ti2a microscope. Data were aggregated from two biological replicates distinguished by shading. Individual biological replicates are also shown in Fig. S1B. (B) Confocal images [*z*-stack maximum-intensity projection (‘Max IP’)] taken on an AXR Nikon microscope of MDCK monolayers fixed and stained for DNA (Hoechst, gray), F-actin (phalloidin, cyan) and α-cat (native GFP fluorescence, green). Overlay images with complementary orthogonal *x-z* views along mitotic cells (dotted yellow lines) show apical extension of the nucleus during mitosis (yellow arrowheads). Scale bars: 10 µm. (C) Schematic of mitotic cells (dark green) on membranes of neighboring cells (light green) during mitotic phases for the α-cat mutants. Mitotic α-cat-KO^GFP-α-cat-4A^ cells exhibited the smallest cell area, suggesting rounding in the *z*-direction. Green arrowheads indicate the *x-z* side view; yellow arrowheads summarize the area quantification differences in A and B.

To address the consequences of α-cat P-linker phosphorylation for mitosis, we restored α-cat CRISPR-knockout (KO) MDCK cells (Quinn et al., 2024) with GFP-tagged α-cat proteins, in which previously mapped phosphorylation sites were blocked or charge-mimicked by amino acid substitutions (α-cat 4A and 4E mutants, respectively) (Fig. 2). Newly confluent MDCK cell monolayers grown on glass coverslips (48 h) were fixed, stained for DNA and imaged to quantify epithelial cell shape changes during established phases of cell division (metaphase, anaphase and telophase), which we reasoned might be altered by the α-cat P-linker modification state. By tracing mitotic cell perimeters (Fig. S1A), we found that cells expressing the α-cat phospho-mimic (4E) protein appeared significantly larger than cells expressing the α-cat phospho-mutant (4A) protein (Fig. 3A, superplot from two biological replicates; Fig. S1B shows biological replicates separately). This apparent difference in mitotic cell area is not due to intrinsic differences in cell size (Fig. S2). Instead, we found that α-cat-4E-restored MDCK cells showed less apical rounding than α-cat wild-type (WT) or α-cat-4A-expressing cells (Fig. 3B,C, orthogonal *x-z* views). Indeed, the apical surface of newly confluent α-cat-4E-expressing epithelial monolayers appeared taut and generally flatter than that of α-cat-WT- or α-cat-4A-expressing cells; conversely, the cortex of mitotic α-cat-4A-expressing cells appeared slack, following the contours of condensed chromosomes and nuclei (Fig. 3B, arrows; Movie 1). These data suggest that full phosphorylation of the P-linker region of α-cat constrains mitotic rounding within the epithelial monolayer and generally promotes epithelial maturation.

### Phospho-mimic α-cat reduces barrier leak during mitosis

**Fig. 4.**
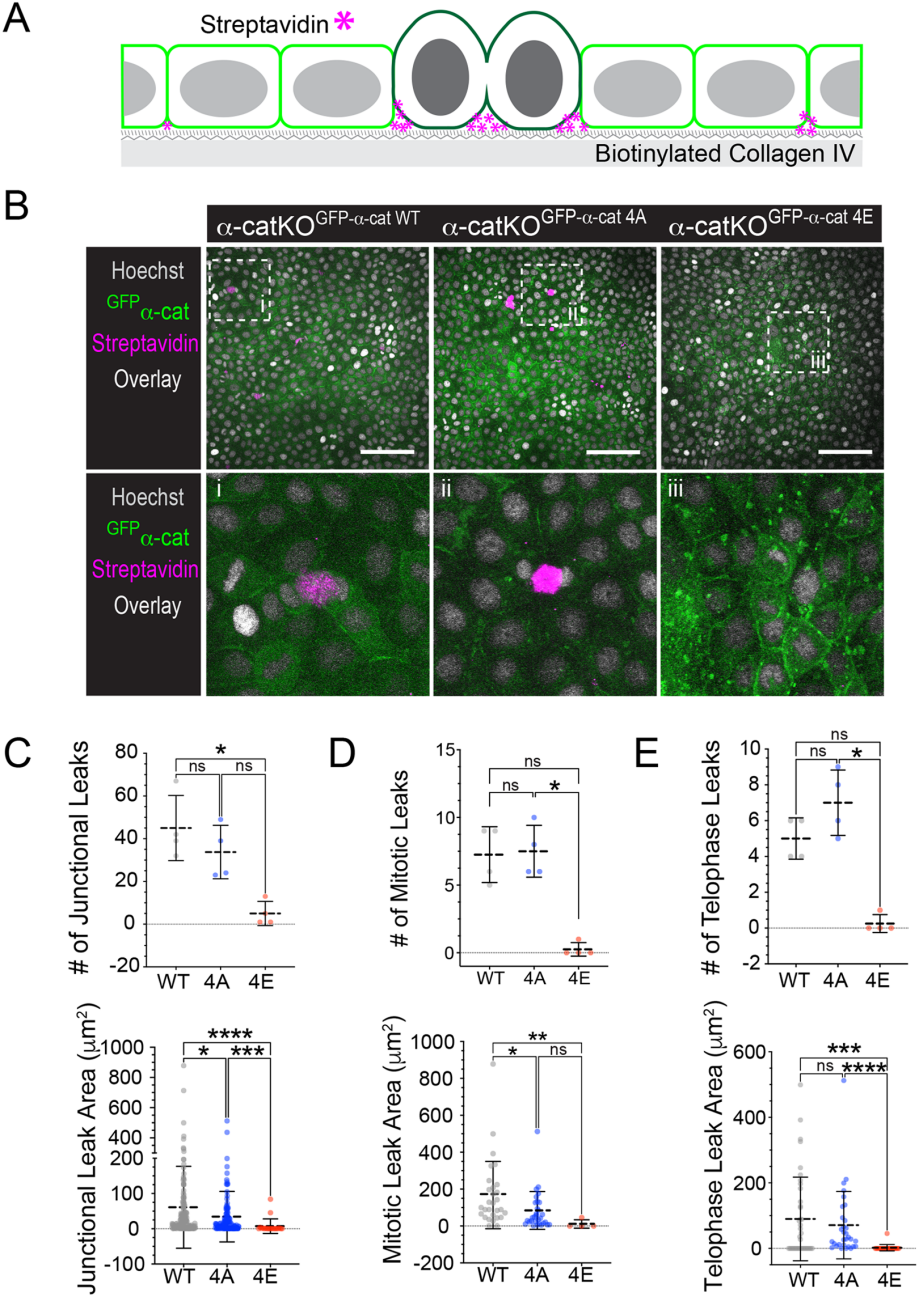
**Phospho-mimic α-cat reduces barrier leak during mitotic rounding compared with α-cat-WT and α-cat-4A.** (A) Schematic of biotin-streptavidin permeability assay, where streptavidin (magenta asterisks) binds to biotinylated collagen IV at barrier leaks during mitosis rounding. (B) Confocal image (*z*-stack maximum-intensity projection of basal region) taken on an AXR Nikon microscope of MDCK cells from permeability assay fixed and stained for DNA (Hoechst, gray), streptavidin binding to biotinylated collagen (magenta) and α-cat (native GFP fluorescence, green). Overlay images show basal biotin-streptavidin interactions in α-cat-KO^GFP-α-cat-WT^ and α-cat-KO^GFP-α-cat-4A^ during telophase (white box insets, i-iii). α-cat-KO^GFP-α-cat-4E^ monolayers showed no substantial streptavidin signal. Scale bars: 100 µm. (C) Quantification of total leak area and number of junctional leaks between α-cat mutants (see Fig. S3A regarding how mitotic versus junctional leaks were distinguished). Data are presented as mean±s.d. (four fields of view) for one biological replicate with significance determined by one-way ANOVA with Kruskall–Wallis test (****P<0.0001; ***P=0.0003; *P=0.0122; ns, not significant). Each symbol reflects leak quantification for a field of view. (D) Quantification of mitotic cell proximal leak area and number of mitotic cell leaks between α-cat mutants. Data are presented as mean±s.d. (four fields of view) for one biological replicate with significance determined by one-way ANOVA with Kruskall–Wallis test (**P=0.0233; *P=0.0028). (E) Quantification of telophase proximal leak area and total number of telophase leaks between α-cat mutants. Data are presented as mean±s.d. (four fields of view) for one biological replicate with significance determined by one-way ANOVA with Kruskall–Wallis test (****P<0.0001; ***P=0.0003; *P=0.0122). An independent experiment showing similarly enhanced leak for α-cat-KO^GFP-α-cat-4A^ cells is presented in Fig. S3B-D.

Mitosis generally relies on actomyosin contractility-dependent rounding to accommodate spindle formation for chromosome segregation and cytokinesis into genetically identical daughters (Taubenberger et al., 2020). Epithelia need to execute these steps in a manner that preserves interactions with neighbors to maintain the barrier, a key function of epithelia across tissue types (Higashi et al., 2016). We wondered, therefore, whether α-cat phosphorylation in the P-linker might limit intercellular junction leak, particularly between mitotic cells and their non-dividing neighbors. We used an established assay to visualize small or transient intercellular leaks, which seeds epithelial cells on a biotinylated collagen matrix at confluent density and reveals monolayer breach via fluorescent dye-conjugated streptavidin (Dubrovskyi et al., 2013; Monster et al., 2021) (schematic in Fig. 4A). We observed many leaks in α-cat-WT- or α-cat-4A-restored MDCK cells, particularly during telophase when actomyosin forces may be peaking. Very few breaks were detected in α-cat-4E-restored MDCK cells (Fig. 4B). Qualitatively, leak size (area) was greater for α-cat-WT- and α-cat-4A- than for α-cat-4E-restored MDCK cells (Fig. 4C-E). These data suggest that full phosphorylation of the P-linker region of α-cat promotes epithelial barrier integrity during mitosis by strengthening interactions between dividing and non-dividing neighbors. α-cat phosphorylation also appeared to play a more general role in epithelial barrier integrity (Fig. S3A, small junction leaks in blue). An independent biological replicate experiment showed similar findings (Fig. S3B-D).

### Phosphorylated α-cat localizes to the apical-most portion of epithelial cell junctions

**Fig. 5.**
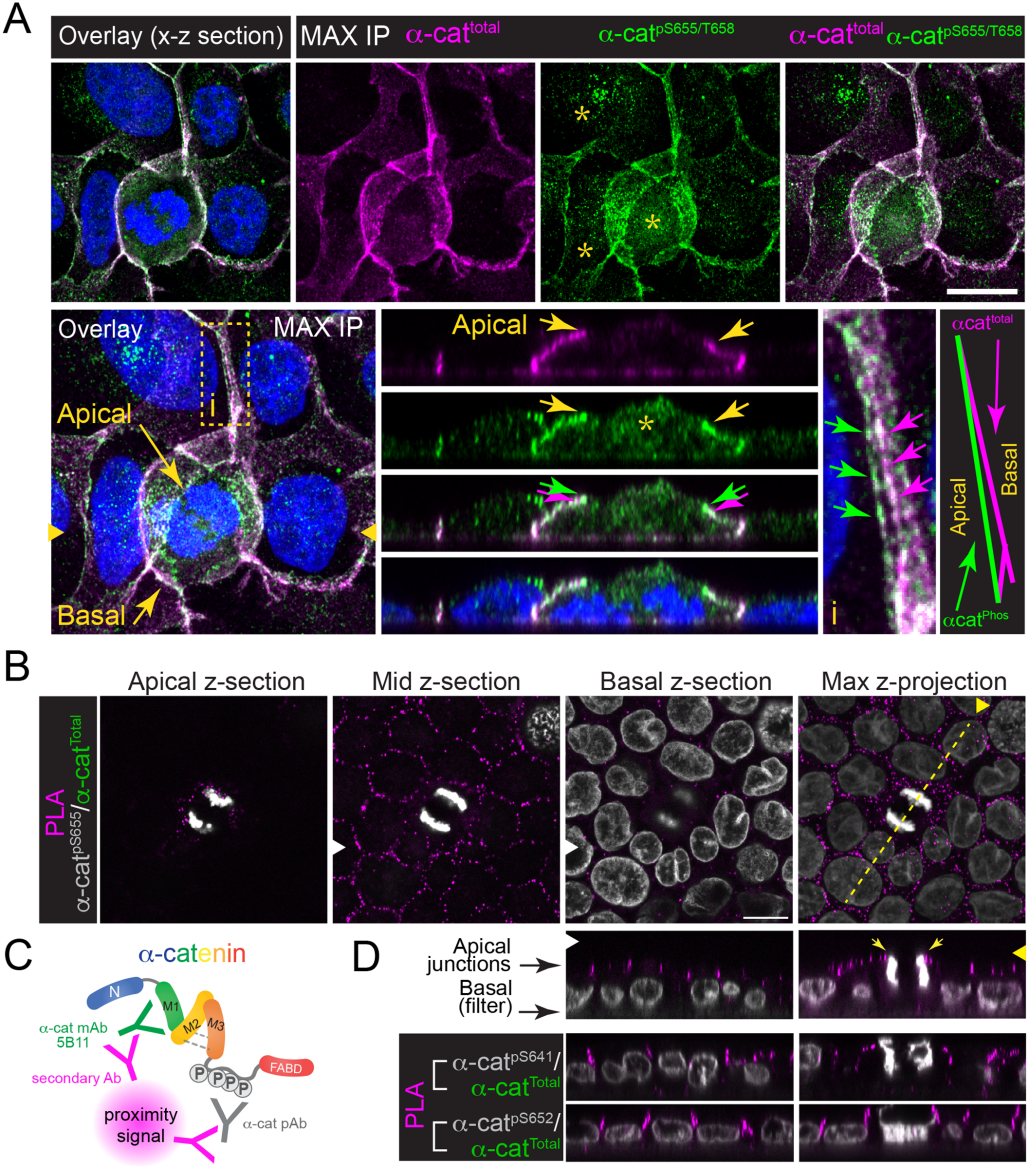
**Phospho-α-cat localizes to the apical-most portion of epithelial cell junctions.** (A) Confocal image [*z*-stack maximum-intensity projection (‘Max IP’)] of MDCK monolayers (glass coverslip grown) fixed and immunostained with antibodies against α-cat (magenta) and α-cat phosphorylated at S655/T658 (green). DNA was stained with Hoechst (blue). Overlay images with complementary orthogonal *x-z* views show pS655/T658 apical junction enrichment (yellow arrows) in both mitotic cells and adjacent cell junctions (yellow box inset, i). Asterisks shows punctate cytoplasmic staining with the anti-pS655/T658 antibody that is likely non-specific. Yellow arrowheads indicate the plane of the *x-y* section (right). Green and magenta arrows indicate pS655/T658 versus total α-cat staining, respectively. (B) Confocal *x-y* sections of α-cat pS655/T658 coincidence detection (magenta spots) using proximity ligation assay (PLA) on filter-grown MDCK cells (10 days). DNA is shown in gray. (C) Schematic of PLA using two antibodies against α-cat. (D) Orthogonal *x-z* sections of α-cat-pS655/T658, α-cat-pS641 and α-cat-pS652 co-incidence detection (magenta spots). The yellow arrowhead pointing to the dotted yellow line in B indicates the plate of the *x-z* section shown in D. Other *x-z* views from pS655/T658/total α-cat (top), pS641/total α-cat (middle) and pS652/total α-cat (bottom) PLA are shown with (right) and without (left) a mitotic cell. Scale bars: 10 µm.

HeLa cell phospho-proteomic and α-cat phospho-antibody immunoblot data revealed that the α-cat P-linker region is maximally phosphorylated during mitosis (Fig. 1) (Dephoure et al., 2008; Olsen et al., 2010). As HeLa cells synchronized in mitosis are released from tissue culture plates after rounding (i.e. double-thymidine block, post-nocodazole mitotic ‘release’ method; Dephoure et al., 2008; Olsen et al., 2010), it is likely that the increase in α-cat phosphorylation occurs within the mitotic cell itself, rather than via neighboring cells (i.e. via a mitotic cell-autonomous versus non-autonomous mechanism). We wondered, therefore, whether we could determine subcellular localizations of phospho-specific forms of α-cat in dividing MDCK cells using available antibodies (Fig. 1). We chose to assess phospho-α-cat localization in MDCK rather than HeLa cells, as the latter are derived from a poorly differentiated adenocarcinoma and not strongly self-adherent (Doyle et al., 1995), although adherens-like structures have been described (Deng et al., 2008; Izawa et al., 2002; Pestonjamasp et al., 1997). Interestingly, antibodies that recognize terminal phosphorylations in the α-cat CK1 sequence – pS655 and T658 (Escobar et al., 2015) – decorated cell-cell junctions of both dividing and non-dividing MDCK cells (Fig. 5; Fig. S4). Confocal imaging showed that antibodies to phospho-α-cat largely overlapped with an antibody that recognizes total α-cat (Fig. 5A, top row). Curiously, optical sections in the *x-z* direction showed that the phospho-α-cat signal appeared to specifically decorate apical junctions (Fig. 5A, magenta/green arrows; see also inset i). As antibodies to α-cat-pS655/T658 also showed an extra-junctional punctate staining pattern (asterisks in Fig. 5A), possibly elevated in mitotic cells, we used a proximity ligation assay (PLA) to validate the localization of phospho-α-cat in MDCK cells (Fig. 5B,C). This method allowed us to use PLA as a ‘coincidence-detection’ system for total and phospho-α-cat, amplifying the cellular localization of phospho-α-cat and reducing impact of antibody cross-reactivity with other possible pS/T epitopes (although we note that the anti-pS655/T568 antibody does not detect cross-reactive bands across a wide molecular mass range by immunoblot analysis, Fig. 2). Although the amplified proximity signal of total α-cat/phospho-α-cat was sparse compared to indirect immunofluorescence methods, this method appeared to selectively detect phospho-α-cat at cell-cell junctions (Fig. 5B,C). Curiously, optical sections in the *x-z* direction showed that the total α-cat/phospho-α-cat proximity signal was at the apical-most portion of cell-cell junctions (Fig. 5D). Proximity detection using antibodies to α-cat pS641 and pS652 showed similar apical bias (Fig. 5D, lower panels). Note that the MDCK monolayer in Fig. 5A was grown on glass, in contrast to filter-grown cells in Fig. 5D, which may contribute to the greater apical enrichment of phospho-α-cat in the latter images. Surprisingly, we saw no obvious increase in proximity-amplified total α-cat/phospho-α-cat signal in dividing versus non-dividing MDCK cells (Fig. 5B,D, yellow arrowheads). These data are in contrast with the increased abundance of phospho-α-cat detected in mitotic or nocodazole-treated HeLa cells (Fig. 1) (Dephoure et al., 2008; Olsen et al., 2010), and suggest that the extent of α-cat phosphorylation may be more related to a feature common to mitotic HeLa cells and MDCK cell monolayers.

## DISCUSSION

Although the cadherin-catenin complex is long known to be required for adherens junction organization and epithelial barrier homeostasis (Gumbiner et al., 1988), we know comparatively less about how and under what conditions the cadherin-catenin complex is regulated. A major paradigm shift in thinking about adherens junction regulation is that the cadherin-catenin complex is mechanosensitive, particularly via its essential actin-binding component, α-cat (Angulo-Urarte et al., 2020). Indeed the actin-binding and middle-domains of α-cat undergo force-dependent unfolding to engage F-actin or various actin-binding effectors (e.g. vinculin), respectively (Barrick et al., 2018; Buckley et al., 2014; Kim et al., 2015; Twiss et al., 2012; Wang et al., 2022; Yao et al., 2014; Yonemura et al., 2010). This allows actomyosin-force dependent strengthening of α-cat binding to actin via direct and indirect mechanisms. Curiously, α-cat is not only regulated by force; α-cat is highly phosphorylated in an unstructured region that links the mechanosensitive middle and actin-binding domains (known as the P-linker region) (Escobar et al., 2015). Although previous *in vitro* kinase assays revealed an elaborate dual-kinase mechanism, wherein phosphorylation at S641 by CK2 effectively primes α-cat for further sequential phosphorylation at S652, S655 and T658 by CK1 (Escobar et al., 2015), the cellular processes and upstream kinase/phosphatase signals that promote α-cat phosphorylation *in vivo* remained unknown.

Here, we leveraged previously published high-throughput phospho-proteomics data (Dephoure et al., 2008) to show that phosphorylation of the P-linker region of α-cat is elevated during mitosis, particularly at previously characterized CK1 sites (Escobar et al., 2015), using HeLa cell synchronized lysates and validated phospho-specific antibodies to α-cat (Cell Signaling Technology). As HeLa cells are cancer-derived and not typically used for studying cell-cell adhesion, we sought to validate the role of α-cat phosphorylation during mitosis in MDCK cells, a longstanding model to study epithelial junctions (Dukes et al., 2011). By reconstituting α-cat CRISPR KO MDCK with WT, phospho-mutant (4A) or phospho-mimic (4E) forms of GFP-α-cat, we show that amino acid charge substitution of the P-linker of α-cat, which aims to mimic the full and persistent phosphorylation of α-cat, constrains mitotic division within the plane of an MDCK epithelial monolayer, limiting intercellular breaks that form between dividing and non-dividing cells. We also observed that α-cat-WT- and phospho-mutant-restored nascent MDCK monolayers appeared generally leakier and less mature than the α-cat phospho-mimic line, with the former showing a more ‘fried egg’ morphology with compliant apical membranes overlying the nucleus. These data suggest that full phosphorylation of the α-cat P-linker region may also be generally required for epithelial monolayer shape transitions that lead to a mature barrier (Cammarota et al., 2024). Overall, although these data are in line with our previous work showing that α-cat phosphorylation contributes to epithelial monolayer adhesive strength and cell-cell coordination during collective migration (using an α-cat shRNA MDCK knockdown/GFP-α-cat reconstitution system; Escobar et al., 2015), they advance an important new concept – α-cat phosphorylation is not simply constitutive, but can increase during mitotic morphogenesis to maintain epithelial barrier function under strain.

We do not yet understand how mitotic signaling causes the upregulation of α-cat phosphorylation at CK1 sites. We previously discovered that the CK1 sites in α-cat are less accessible to in-solution phosphorylation by CK1 in full-length α-cat compared with a fragment comprising only the C-terminal half of α-cat (fig. 2G-H of Escobar et al., 2015). This raises the possibility that α-cat binding to actin or increased actomyosin contractility associated with mitosis might favor α-cat P-linker unfolding and kinase accessibility. However, we cannot exclude the possibility that mitosis upregulates other kinases or inhibits phosphatases that target α-cat at S652, S655 and T658.

We also do not fully understand how α-cat phosphorylation reinforces epithelial barriers during cell division. Recent studies implicate vinculin, an α-cat homologue and mechanosensitive binding partner as a key adherens junction reinforcer during cell division (Higashi et al., 2016; Monster et al., 2021). In *Xenopus*, vinculin becomes enriched along the cytokinetic furrow, coincident with a reduction in cadherin/catenin mobility (Higashi et al., 2016). As loss of vinculin or its coupling to actin enhances the rate of furrow ingression and tight junction leak (Higashi et al., 2016; van den Goor et al., 2024), it appears that the speed of mitosis/cytokinesis must be carefully controlled by the cadherin-catenin complex (Goldbach et al., 2010; Padmanabhan et al., 2017) to ensure epithelial barrier maintenance during cell division. Evidence from MDCK cells suggests that mitotic force-dependent α-cat-unfolding and recruitment of vinculin appears to be asymmetric, requiring reinforcement of adherens junctions by vinculin in cells surrounding rather than within the mitotic cell (Monster et al., 2021). Given these data, it is attractive to speculate that α-cat phosphorylation-dependent epithelial barrier reinforcement during cell division may be due, at least in part, to enhanced vinculin recruitment. However, as we previously found that an α-cat phospho-mimic mutant incapable of binding vinculin could not reverse cell-cell cohesive behaviors enhanced by phosphorylation (Escobar et al., 2015), α-cat phosphorylation likely impacts α-cat structure and function more broadly, and beyond simply recruiting vinculin (J.M.Q. and C.J.G., unpublished data).

Evidence that a mitotic cell rounding against its neighbor can lead to adherens junction asymmetry (Monster et al., 2021) inspired us to look closely at where phospho-α-cat is localized in dividing MDCK cells. Although phosphorylation of the P-linker region of α-cat was clearly elevated in mitotic HeLa cell lysates, we saw no clear increase in phospho-α-cat detection along the dividing/non-dividing MDCK adherens junction. Instead, we found that phospho-α-cat appeared localized to adherens junctions more generally, and notably the apical-most region of adherens junctions known as the zonula adherens (Mangeol et al., 2024; Mooseker et al., 1983). Similar immunofluorescence analysis in HeLa cells was not possible, possibly because this cancer-derived cell line is known to make only weak spot-like adherens junctions (Deng et al., 2008; Izawa et al., 2002; Pestonjamasp et al., 1997) (not shown). We speculate, therefore, that full phosphorylation of the α-cat P-linker region may depend on a property common to mitosis and zonula adherens junctions, such as a reliance on actomyosin-based contractility (Murrell et al., 2015; Nyga et al., 2023; Sorce et al., 2015; Yap et al., 2018). Indeed, evidence that nocodazole-treated HeLa cells also show similar increase in α-cat phosphorylation at pS652 and pS655/T658 CK1 sites, where nocodazole is known to induce a hypercontractile actomyosin state (Chang et al., 2008), suggests that α-cat phosphorylation is not simply cell cycle dependent. It is also worth noting that the P-linker is highly phosphorylated in α-cat isoforms expressed in cell types that are non-dividing (e.g. αT-cat in cardiomyocytes) or lacking tight junctions (e.g. αN-cat in neurons) (Ballif et al., 2004; Reitz et al., 2023). Thus, regulation of the P-linker of α-cat appears important across all α-cat isoforms and cell-specialized adherens junction organizations.

In summary, these data suggest that full phosphorylation of the P-linker region of α-cat promotes epithelial barrier integrity during mitosis by strengthening interactions between dividing and non-dividing neighbors. Along with previously published phospho-proteomic data sets showing that major scaffold components of adherens junctions, tight junctions and desmosomes are differentially phosphorylated during mitosis (Dephoure et al., 2008; Olsen et al., 2010) (Table 1), we reason that epithelial cell division may be a tractable system to understand how junction complexes are coordinately regulated.

**Table 1.** Differential phosphorylation of major scaffold components of adherens junctions, tight junctions and desmosomes in S phase and mitosis

Gene (protein)	Number of phospho-sites in HeLa cells	
	S phase	Mitosis
**Adherens junctions**		
*CTNNA1* (α-catenin)	5	5*
*CTNNB1* (β-catenin)	0	**2**
*CTNND1* (p120^ctn)^	5	**9**
*ARVCF* (δ-catenin)	1	**2**
*PKP4* (plakophilin 4/p0071)	1	**11**
*MLLT4*/*AFDN* (afadin)	6	**10**
*VIN* (vinculin)	1	**2**
**Tight junctions**		
*TJP1* (ZO-1)	6	**22**
*TJP2* (ZO-2)	17	**25**
*TJP3* (ZO-3)	3	**4**
*PARD3* (Par3)	4	**9**
*CGN* (Cingulin)	5	5*
*CLDN12* (Claudin 12)	0	**2**
*F11R* (Jam1)	**6**	2
*MARVELD2* (tricellulin)	**2**	0
*NF2* (merlin)	**1**	0
*JUB* (ajuba)	**1**	0
**Desmosomes**		
*DSG2* (desmoglein)	1	**2**
*DSP* (desmoplakin)	**29**	19
*PKP2* (plakophilin 2)	**6**	3
*PKP3* (plakophilin 3)	**5**	4

Data selected from table S1 of Dephoure et al. (2008). Numbers in bold within junction systems indicate more phosphorylation in S or mitotic phase. *Some phospho-sites are different between S and mitotic phase; others are increased in the mitotic phase.

## MATERIALS AND METHODS

Key resources are provided in Table S1.

### Plasmid constructs

N-terminally GFP-tagged αE-catenins were synthesized by VectorBuilder using a dimerization-disrupted mEGFP (A206K) in third-generation lentiviral vectors with components pLV[Exp]-CMV>mEGFP-αE-catenin EF1A(core)>Puro. Lentivirus packaging (psPAX2, #12260) and envelope (pMD2.G, #12259) plasmids were purchased from Addgene. Previously established α-cat phospho-sites S641, S652, S655 and T658 (Escobar et al., 2015) were changed to alanine (α-cat-4A mutant, which prevents phosphorylation) or glutamate (α-cat-4E mutant, which aims to mimic the phosphate charge).

### Cell culture and stable cell line selection

MDCK II cells were maintained in Dulbecco's modified Eagle's medium (DMEM, Corning), containing 10% fetal bovine serum (FBS, R&D Systems), 100 U/ml penicillin and 100 µg/ml streptomycin (Corning). α-cat/*Ctnna1* knockout MDCK clone 2.2 was generated using CRISPR-Cas9 system as described in Quinn et al. (2024). For lentivirus production, 293T cells (GeneHunter) were transfected with 8 µg expression vector (VectorBuilder), 6 µg psPAX2 and 2 µg pMD2.G using TransIT transfection reagent (Mirus). The viral supernatant was collected 48 and 72 h after transfection, passed through a 0.45 µm filter and supplemented with 1 µl/ml polybrene (Sigma-Aldrich). To generate stable GFP-α-cat lines, MDCK α-cat KO cells were transduced for 6 h at 37°C on 10 cm plates with 2 ml prepared viral supernatant. Cells were selected in culture medium containing 5 µg/ml puromycin, then expression matched for GFP using a FACSMelody 3-laser sorter (BD Biosciences).

### Antibodies

The following primary antibodies were used for immunoblot analysis: mouse monoclonal anti-α-cat (15D9, Enzo Life Sciences, ALX-804-101, 1:200-1:1000), hybridoma mouse anti-α-cat (5B11, 1:2 dilution, from Arnold J. Levine, Princeton University; Daugherty et al., 2014) and polyclonal rabbit anti-GFP (1:1000, A11122, Invitrogen). Secondary antibodies for western blotting included HRP-conjugated goat anti-mouse and anti-rabbit antibodies (1:2500, Abcam, ab139417) or fluorescently labeled donkey anti-mouse and anti-rabbit antibodies (680RD or 800RD, LiCor Biosciences). Cell Cycle and Apoptosis Western Blot Cocktail (actin, Cdk2 pY15, histone H3 pS10) was provided by Abcam (ab139417, diluted 1:250). For immunofluorescence analysis, we used: hybridoma mouse anti-α-E-catenin (1:2, 5B11), rabbit anti-α-cat-pS652 (1:200, 13061, Cell Signaling Technology), rabbit anti-α-cat-pS655/T658 (1:200, 13231, Cell Signaling Technology), rabbit anti-α-cat-pS641 (1:200, 11330, Signalway) and Alexa Fluor 488 phalloidin (1:2000, A12379, Invitrogen). Secondary antibodies for immunofluorescence included IgG Alexa Fluor 488- or 568-conjugated goat anti-mouse or anti-rabbit antibodies (Invitrogen). Criterion gradient gels (4-20% acrylamide) were used to separate proteins by SDS-PAGE. Immunoblots were imaged and the band intensities quantified using a LI-COR Odyssey Imaging system.

#### HeLa lysates

Our independent validation of the HeLa phospho-proteomic data (Fig. 1A) was carried out using two different HeLa cell cycle-synchronized lysates (Abcam, ab136811; lot numbers K and M from 2017 and 2019, respectively) (Fig. 1B). For quantification, we present the data as fold increase over the asynchronized HeLa control condition. Quantification was carried out using the LI-COR densitometry tool. Phospho-α-cat and total α-cat integrated band densities were background subtracted and ratioed (phospho-α-cat signal/total α-cat signal), normalized to the control (asynchronized HeLa condition) and superplotted in Prism. Nocodazole-treated (W09-001-A81) and control (W09-000-364) HeLa cell lysates were purchased from Rockland Immunochemicals.

### Immunofluorescence and imaging

Cells were grown on glass coverslips (300,000 cells/well of six-well plates) or Transwell filters (BD Biosciences,353494) for 10 days as indicated, fixed in 4% paraformaldehyde (Electron Microscopy Services, Hatfield, PA, USA) for 15 min, quenched with glycine, permeabilized with 0.3% Triton X-100 (Sigma-Aldrich), and blocked with normal goat serum (Sigma-Aldrich). Primary and secondary antibody incubations were performed at room temperature for 1 h, interspaced by multiple washes in PBS, and followed by mounting coverslips in ProLong Gold fixative (Life Technologies). Images of mitotic GFP-α-cat-WT-, -4A- and -4E-expressing MDCK cell monolayers (Fig. 3; Figs S1 and S4) were captured with a Nikon Ti2 (B) widefield microscope (DS-Qi2 camera, 20× air objective) using NIS Elements software. Confocal *z*-stack (0.3 µm step size) images (Figs 2 and 5) were captured using a Nikon AXR microscope with GaAsP detectors and equipped with a 95B prime Photometrics camera and a Plan-Apochromat 40× objective.

### PLA

MDCK cells were plated at 50,000 cells/well and cultured on 0.4 µm pore 12-well Transwell filters (BD Biosciences, 353494), for 10 days in DMEM containing 10% FBS as described above. The medium was aspirated and cells were rinsed in PBS containing calcium and magnesium (Corning, 21-030-CV), followed by fixation in ice-cold anhydrous methanol (Sigma-Aldrich, MX0487-6) for 15 min. Cells were rinsed three times in PBS (Sigma-Aldrich, D5652) and permeabilized in PBS containing 0.3% Triton X-100 (Sigma-Aldrich, T8787) for 30 min at room temperature. Cells were blocked in Duolink Blocking Solution (DUO82007, Sigma-Aldrich) for 60 min at 37°C. Antibodies were diluted in Duolink Antibody Diluent (DUO82008, Sigma-Aldrich): hybridoma mouse anti-α-E-catenin (1:2, 5B11), rabbit anti-α-cat-pS652 (1:200, 13061, Cell Signaling Technology), rabbit anti-α-cat-pS655/T658 α-cat (1:200, 13231, Cell Signaling Technology) and rabbit anti-α-cat-pS641 (1:200, 11330, Signalway). Cells were incubated in primary antibodies for 60 min at room temperature, followed by two washes in Duolink 1× Wash Buffer A for 5 min at room temperature. Duolink *In Situ* PLA Probe Anti-Rabbit PLUS (DUO92002, Sigma-Aldrich) and Duolink *In Situ* PLA Probe Anti-Mouse MINUS (DUO92004, Sigma-Aldrich) were diluted 1:5 in Duolink Antibody Diluent, and cells were incubated with probes in a pre-heated humidity chamber for 1 h at 37°C. Cells were washed twice in Duolink 1× Wash Buffer A for 5 min at room temperature. Duolink Ligase was diluted 1:40 in 1× Duolink Ligation Buffer, and cells were incubated with Duolink Ligase in a pre-heated humidity chamber for 30 min at 37°C. Cells were washed twice in Duolink 1× Wash Buffer A for 5 min at room temperature. Amplification Polymerase RED (DUO92008, Sigma-Aldrich) was diluted 1:80 in Duolink 1× Amplification Buffer, and cells were incubated with the polymerase in a pre-heated humidity chamber for 100 min at 37°C. Cells were washed twice in Duolink 1× Wash Buffer A for 10 min and once in 0.01× Wash Buffer B for 1 min at room temperature. Cells were then incubated with Hoechst diluted 1:10,000 in 0.01× Wash Buffer B for 5 min at room temperature, followed by a rinse in 0.01× Wash Buffer B. Transwell filters with cells were cut from the cup and mounted to slide under #1.5 coverslips using ProLong Gold AntiFade Mountant (Thermo Fisher Scientific, P36934). Slides were allowed to dry overnight at room temperature and stored at −30°C until imaging.

### Epithelial permeability immunofluorescence assay

Glass-bottomed dishes (Falcon) were coated with 1 mg/ml Collagen IV (C5533, Sigma-Aldrich) for 30 min at 37°C. Then, dishes were biotinylated with EZ-Link-NHS-LC-Biotin (21336, Thermo Fisher Scientific) at 1.5 mg/ml overnight at 4°C. MDCK α-cat knockout cells expressing GFP-α-cat-WT, GFP-α-cat-4A or GFP-α-cat-4E were seeded at a density of 300,000 cells/well (six-well plate) and cultured for 48 h to develop a nascent epithelial monolayer. Cells were washed with ice-cold PBS-Ca^2+^/Mg^2+^, treated with 25 µg/ml streptavidin conjugated with Alexa Fluor 568 (S11226, Thermo Fisher Scientific) for 30 min at 4°C, rinsed with PBS, fixed and mounted for imaging on a Nikon Ti2 (B) widefield microscope (DS-Qi2 camera, 20× air objective).

### Image analysis and barrier leak quantification

To quantify the contribution of α-cat phosphorylation to mitotic rounding, cell area was quantified from maximum-intensity projection images in FIJI (ImageJ). Mitotic phases were determined from the stereotypical organization of condensed chromosomes using the Hoechst DNA stain [e.g. metaphase (chromosomes condensed and centrally aligned), anaphase (separation of condensed chromosomes) and telophase/cytokinesis (decondensed chromosomes and cytokinetic furrow)]. The area was measured in regions of interest through hand tracing of cell junctions from the GFP-α-cat signal (Fig. S1). To compare barrier function of GFP-α-cat WT-, phospho-mutant- or phospho-mimic-restored MDCK cells, junction leak signal (streptavidin conjugated with Alexa Fluor 568) was quantified, focusing on the monolayer basal/glass surface. For data in Fig. 3 and Fig. S3, leak number (spots) were manually counted; Area was measured in FIJI by hand tracing the biotin-streptavidin signal. All quantifications show mean±s.d. and significance was determined using GraphPad Prism. Technical versus biological replicates are transparently shown in each figure and legend.

## Supplementary Material

10.1242/biolopen.061726_sup1 Supplementary information
