# Supplementary material for: α-catenin phosphorylation is elevated during mitosis to resist apical rounding and epithelial barrier leak
Source: Biol Open. 2025 Jan 8;14(1):bio061726. doi: 10.1242/bio.061726 (PMC11744050; doi:10.1242/bio.061726)
Supplement: Supplementary information [file biolopen-14-061726-s1.pdf]

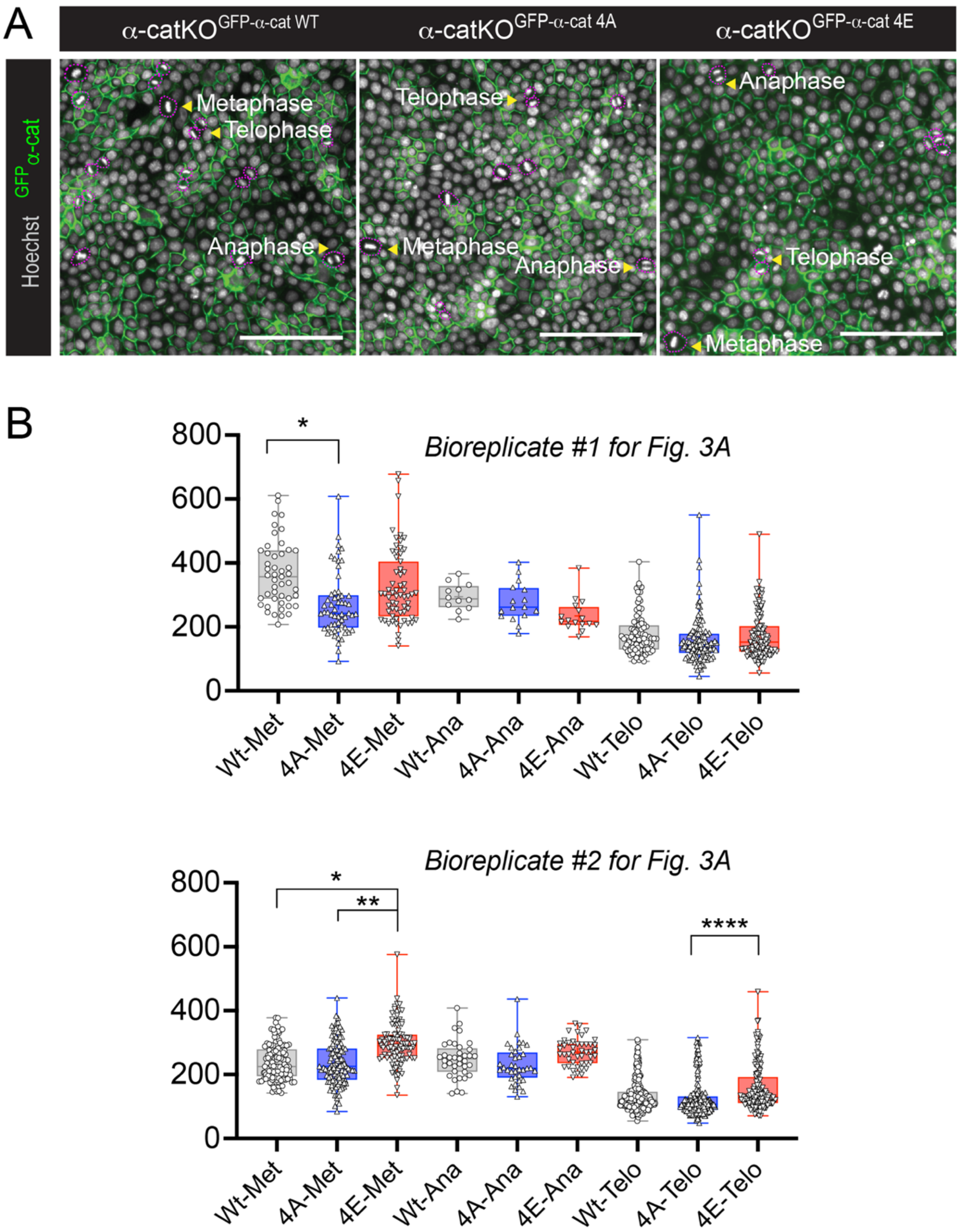
A
α-catKO GFP-α-cat WT
α-catKO GFP-α-cat 4A
α-catKO GFP-α-cat 4E
Hoechst GFPα-cat
Metaphase
Telophase
Anaphase
B
Bioreplicate #1 for Fig. 3A
Bioreplicate #2 for Fig. 3A
800
600
400
200
0
Wt-Met
4A-Met
4E-Met
Wt-Ana
4A-Ana
4E-Ana
Wt-Telo
4A-Telo
4E-Telo

Fig. S1. Tracings of dividing MDCK cells.

[Related to Fig. 3] **(A)** Nikon Ti2 Widefield images (z-stack maximum intensity projection of basal region) of MDCK fixed and immuno-stained for α-cat (native GFP, green) and Hoechst (gray). Overlay image with cell area hand tracing (dotted magenta) shows criteria for differentiating cell division stage (yellow arrowheads). Scale bar = 100μm. **(B-C)** Quantification of cell area (microns^2) of α-cat cell lines during mitotic phases. Data presented as mean ±SD with significance for each biological replicate. Image captured using a 20x objective Nikon Ti2a microscope. In both experiments, the α-cat phospho-mutant mitotic cells display smaller area in comparison with α-cat WT or 4E cells, where statistical significance is met for the α-cat 4A/WT metaphase comparison in one experiment (* by ANOVA with Kruskal-Wallis test, $p = 0.0220$) versus α-cat 4A/4E in the second experiment (** by ANOVA with Kruskal-Wallis test, $p = 0.0025$). The α-cat 4A/4E telophase comparison in the second experiment is highly significant (****) by ANOVA with Kruskal-Wallis test, $p < 0.0001$).

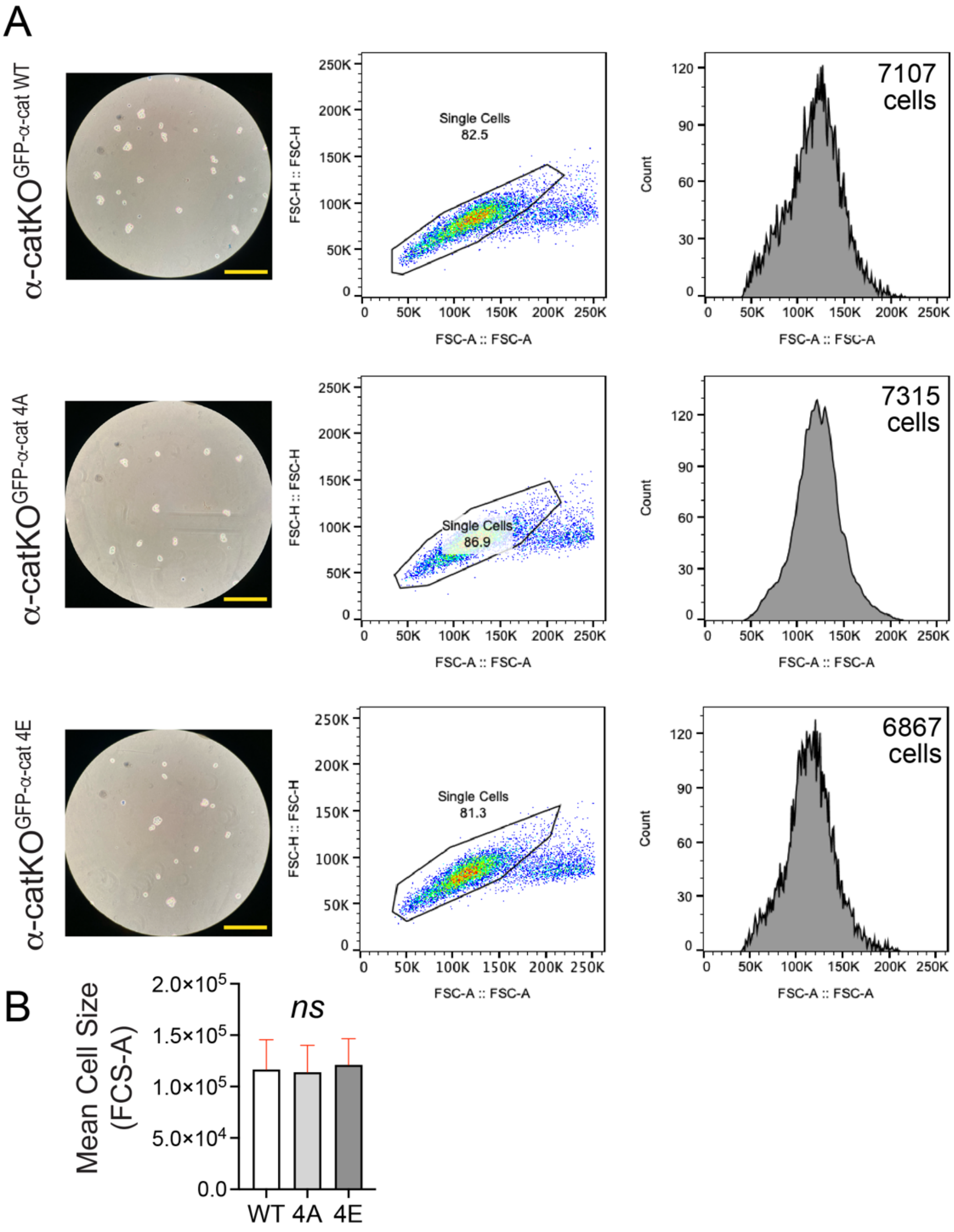

Fig. S2. α-cat WT and phospho-mutant cell sizes are not intrinsically different. *[Related to Fig. 3]* **(A)** Brightfield images taken on Nikon Eclipse TS100 with phone camera of GFP-α-cat WT and mutant-restored MDCK cells after trypsinization. Scale bar = 0.25mm. Cell size quantification of GFP-α-cat-positive cells by flow cytometry. Single cells were gated for each α-cat construct by comparing Forward Scatter Height by Forward Scatter Area in using FlowJo. Histograms show mean fluorescence scatter-area (FCS-A) for each cell line (~8K cells/events quantified). **(B)** Graph shows mean +/- SD with no significant difference in cell size across α-cat WT, 4A or 4E MDCK populations.

Biology Open • Supplementary information

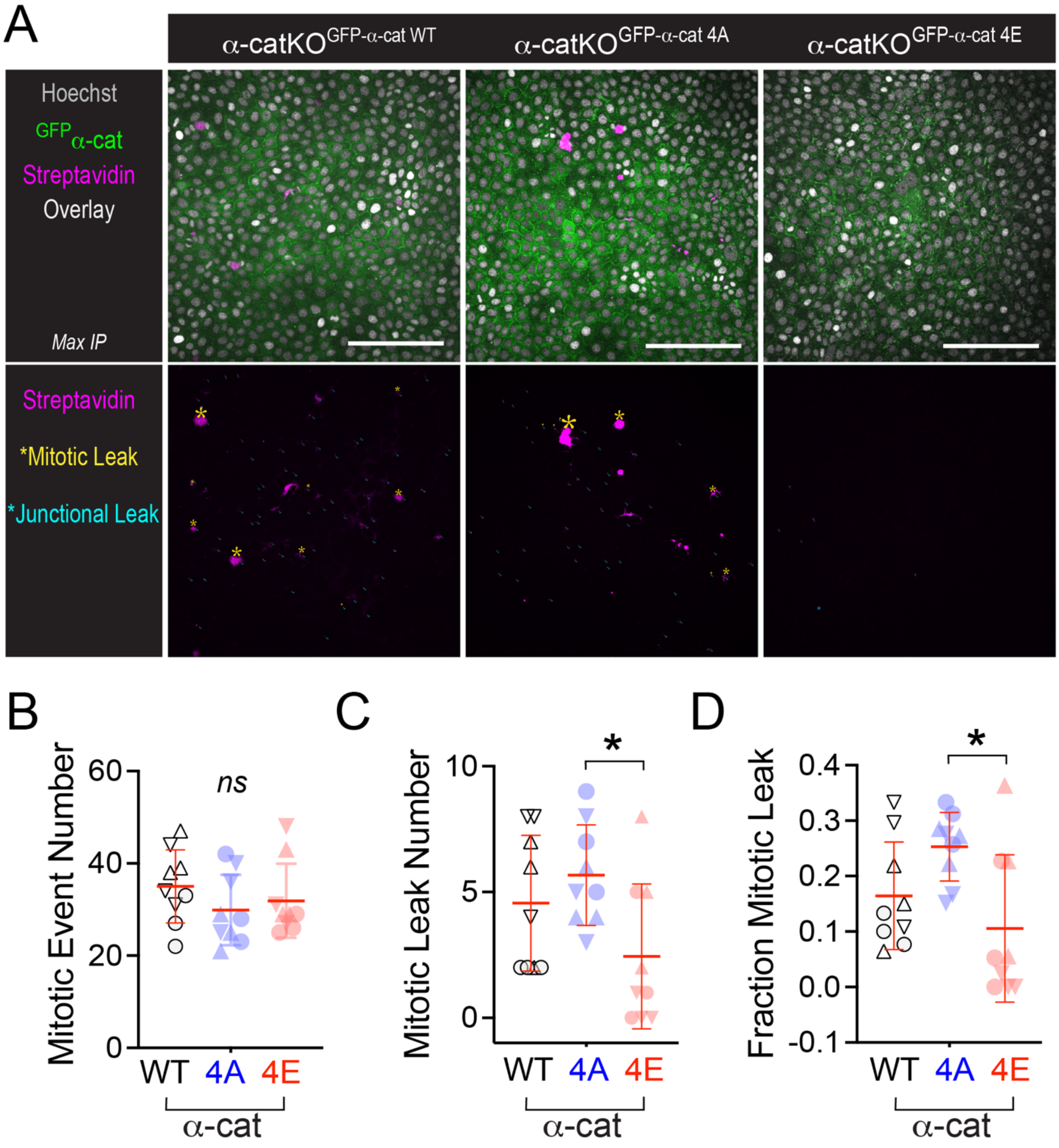

Bioreplicates for Fig. 4

Biology Open • Supplementary information

Fig. S3. Phospho-mimic α-cat reduces barrier leak during mitotic rounding.

[Related to Fig. 4] **(A)** Confocal image (z-stack maximum intensity projection of basal region) taken on Nikon AXR microscope of MDCK permeability assay fixed and immuno-stained for α-cat (native GFP, green), Hoechst (gray), and streptavidin (magenta). Hand tracing of mitotic leaks (yellow asterisk) and junctional leaks (tiny blue asterisks) showed reduced barrier leak in α-cat-KO$^{GFP\text{-}\alpha\text{-}cat\ 4E}$ relative to α-cat-KO$^{GFP\text{-}\alpha\text{-}cat\ WT}$ or α-cat-KO$^{GFP\text{-}\alpha\text{-}cat\ 4A}$ nascent monolayers. Scale bar = 100µm. **(B)** Mitotic Cell Number, **(C)** Mitotic Leak Number and (**D**) Fraction Mitotic Leak (former/latter) with adjacent streptavidin-Alexa-568 signal as evidence of junctional leak. Data quantified from triplicate plating of cells onto biotinylated collagen coverslips x 3 FOVs (~1000 cells/field of view), where distinct symbol shapes reflect data quantified from different coverslips. Data presented as mean ±SD with significance by ANOVA *(p = 0.034 for Mitotic Leak Number and p = 0.0137 for Fraction Mitotic Leak).

Biology Open • Supplementary information

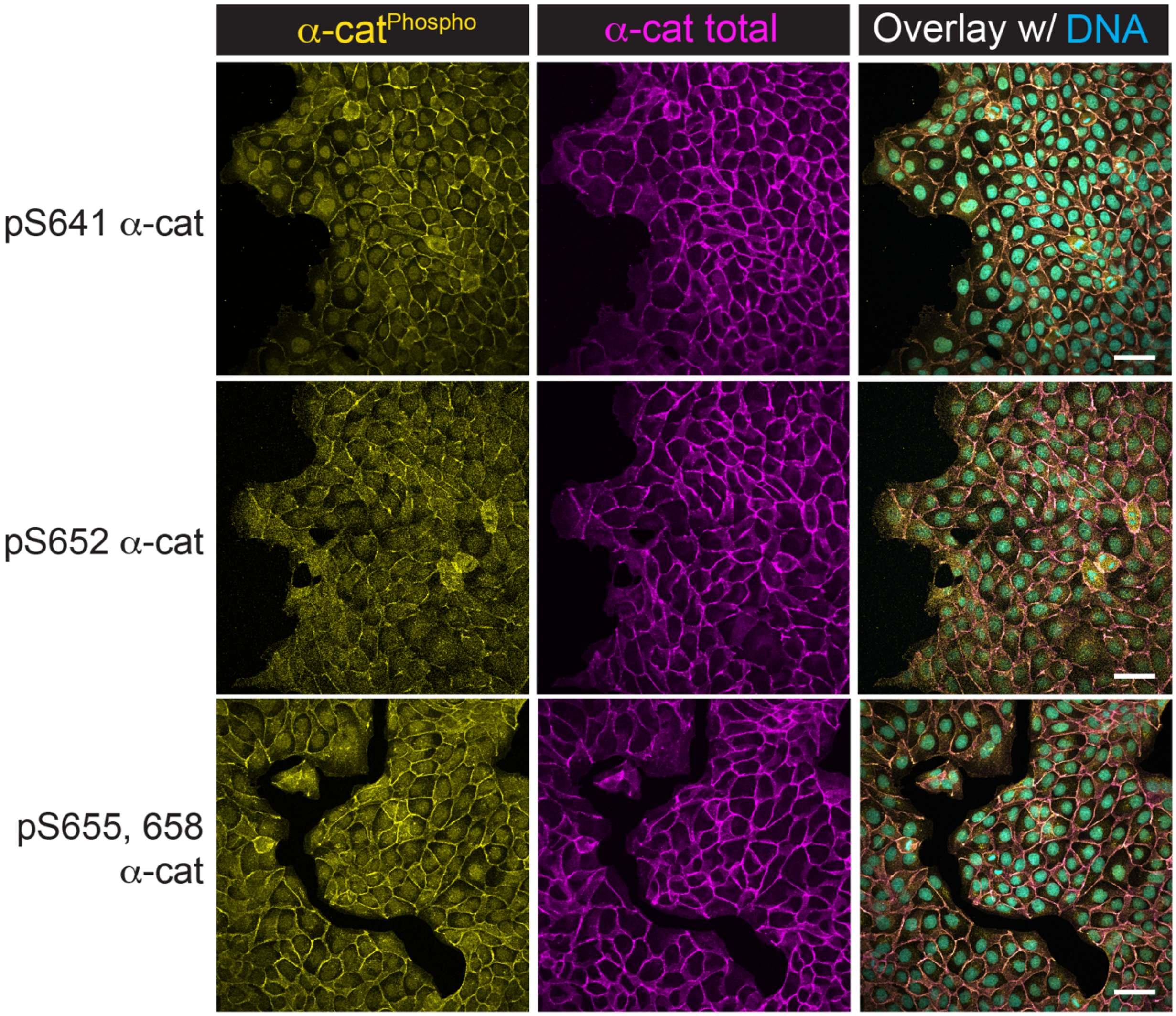

Fig. S4. Phospho-α-cat localizes to epithelial cell junctions.

Confocal images of MDCK monolayer (glass coverslip grown) fixed and immuno-stained with antibodies to α-cat (magenta) and α-cat phosphorylated at S641, S652 and S655/T658 (yellow). DNA stained with Hoechst (cyan). Individual and overlay image shown Scale bar = 50μm.

Table S1. Key Resources Table

REAGENT or RESOURCE	SOURCE	IDENTIFIER
Antibodies		
α-catenin 15D9 (1:200-1:1000, Immunoblot, IB)	Enzo Life Sciences	ALX-804-101
α-catenin 5B11 (1:2, Immunofluorescence, IF)	Hybridoma	Arnold J. Levine, Princeton U.
α-catenin pS641 (1:200, IF; 1:1000, IB)	Signalway	11330
α-catenin pS652 (1:200, IF; 1:1000, IB)	Cell Signaling	13061
α-catenin pS655, 658 (1:200, IF; 1:1000, IB)	Cell Signaling	13231
Alexa Fluor 568 Phalloidin (1:50, IF)	Invitrogen	A12380
Alexa Fluor 488 Phalloidin (1:50, IF)	Invitrogen	A12379
Cell Cycle and Apoptosis Western Blot Cocktail (Actin, Cdk2 pTyr15, Histone H3 pSer10) (1:250)	Abcam	ab139417
GFP (1:1000, IB)	Invitrogen	A11122
Streptavidin, Alexa Fluor 568 (1:500, IF)	ThermoFisher Scientific	S11226
Tubulin (1:2000, IB)	Sigma	T4026
Goat-anti-mouse 488 (1:300, IF)	Invitrogen	A11001
Goat-anti-rabbit 488 (1:300, IF)	Invitrogen	A11008
Goat-anti-mouse 568 (1:300, IF)	Invitrogen	A11004
Goat-anti-rabbit 568 (1:300, IF)	Invitrogen	A11011
Donkey anti-Mouse 680 (1:5000, IB)	LiCORbio	925-68072
Donkey anti-Rabbit 800 (1:5000, IB)	LiCORbio	925-32213
Chemicals, Peptides, and Recombinant Proteins		
Collagen IV	Sigma-Aldrich	C5533
Dulbecco's Modification of Eagle's Medium (DMEM) with 4.5 g/L glucose, L-glutamine, & sodium pyruvate	Corning	10-013-CV
Dulbecco's Phosphate – Buffered Saline (PBS), 1x, without calcium & magnesium	Corning	21-031-CV
Dulbecco's Phosphate – Buffered Saline (PBS), 1x, with calcium & magnesium	Corning	21-030-CM
EZ-Link-NHS-LC-Biotin	ThermoFisher Scientific	21336
Fetal Bovine Serum	BioTechne	S11510
HeLa Cell Cycle Lysates (Lot 'K', 2017; Lot 'M', 2019)	Abcam	ab136811 **Discontinued*
HeLa Cell Lysate	Rockland	W09-000-364
HeLa Cell Lysate (nocodazole treated)	Rockland	W09-001-A81
Penicillin Streptomycin Solution, 50x	Corning	30-001-CI
0.25% Trypsin, 2.21 mM EDTA, 1X [-] sodium bicarbonate	Corning	25-053-CI
Hoechst 33342	ThermoFisher Scientific	62249
ProLong Gold Antifade Mountant	ThermoFisher Scientific	P36934
Critical Commercial Assays		
Duolink In Situ Red Starter Kit Mouse/Rabbit	Millipore-Sigma	Cat #DUO92101-1KT
Duolink In Situ Detection Reagents Red	Millipore-Sigma	Cat #DUO92008
Duolink In Situ PLA Probe Anti-Mouse MINUS	Millipore-Sigma	Cat #DUO92004
Duolink In Situ PLA Probe Anti-Rabbit PLUS	Millipore-Sigma	Cat #DUO92002

Biology Open • Supplementary information

Duolink In Situ Wash Buffers Fluorescence	Millipore-Sigma	Cat #DUO82049
Madin-Darby canine kidney II cells	Gumbiner Lab (1996)	Heidelberg strain (Kai Simmons Lab)
Software and Algorithms		
FIJI/ImageJ (version: 2.1.0/1.53c)	Schneider, CA et al., 2012	https://imagej.nih.gov/ij/
GraphPad Prism		https://www.graphpad.com/scientific-software/prism/
Oxford Instruments Imaris (version 10.1)		https://imaris.oxinst.com/
Other		
AXR Confocal	Nikon Instruments	Galvano scanning, GaAsP detectors
Axioplan2 Epifluorescence Microscope	Zeiss; 20x objective (Air)	AxioCAM HR Camera with AxioVision 4.8 software
Ti2 (B) Widefield Microscope	Nikon Instruments	Wide-field Nikon DS-Qi2 Camera, 20x air objective, NIS Elements
Nikon Eclipse TS100	Nikon Instruments	10x objective, Apple iPhone15 camera
Plasmids		
Monomeric GFP-αE-catenin	VectorBuilder	Human α-Ecat sequence/*CTNNA1; first used in Quinn et al., 2024*
Monomeric GFP-αE-catenin P-linker 4A	VectorBuilder	$S_{641}S_{652}S_{655}T_{658} \rightarrow A_{641}A_{652}A_{655}A_{658}$
Monomeric GFP-αE-catenin P-linker 4E	VectorBuilder	$S_{641}S_{652}S_{655}T_{658} \rightarrow E_{641}E_{652}E_{655}E_{658}$

Biology Open • Supplementary information

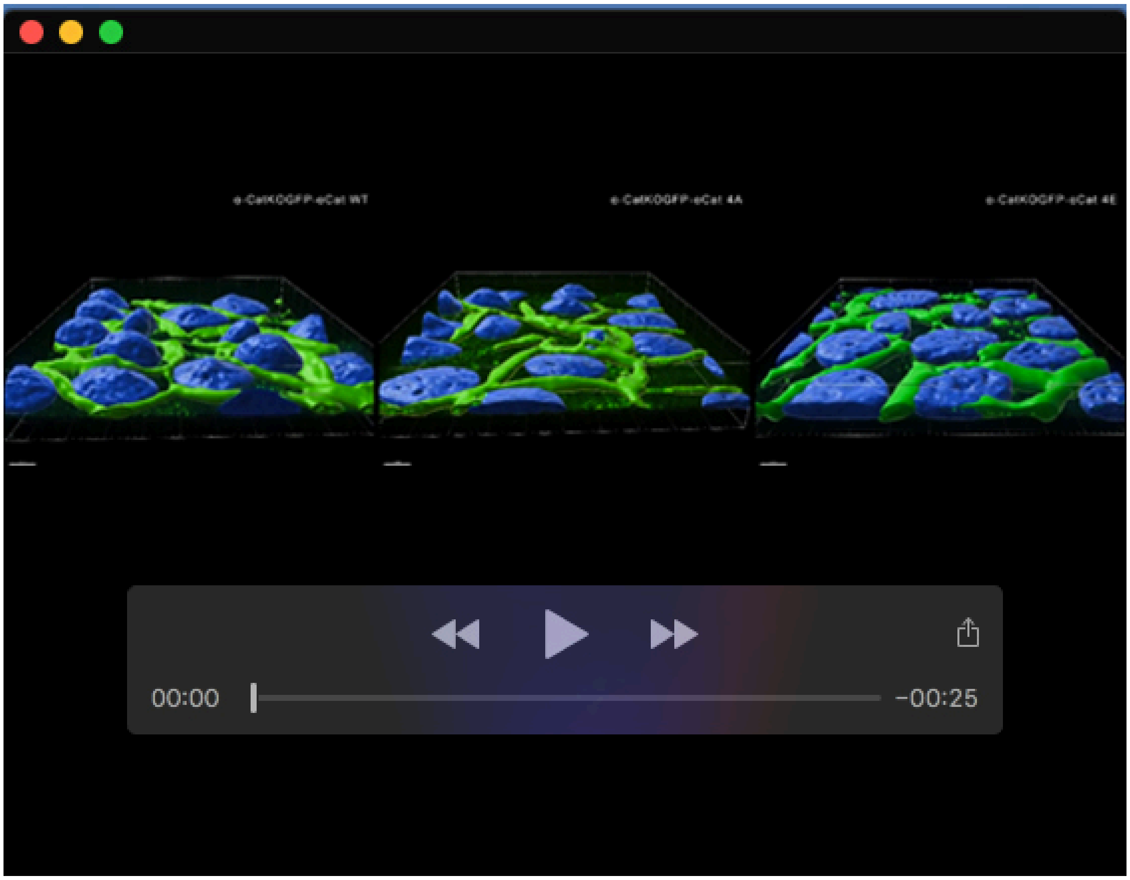

Movie 1. Phospho-mimic α-cat restrains mitotic rounding.

[Related to Fig. 3] Confocal images taken on AXR Nikon microscope of MDCK monolayer fixed and immuno-stained with antibodies to α-cat (green). DNA stained with Hoechst (blue). 4D image visualization on Imaris AI Microscopy Image Analysis Software was threshold, gated for voxels, and surface detail set between 0.2-0.5. Overlay 4D image analysis shows apical extension of nucleus during mitosis. Scale bar = 5μm.

Biology Open • Supplementary information
